# The ratio of 12α to non-12-hydroxylated bile acids reflects hepatic triacylglycerol accumulation in high-fat diet-fed C57BL/6J mice

**DOI:** 10.1038/s41598-022-20838-9

**Published:** 2022-10-06

**Authors:** Wakana Iwasaki, Ryo Yoshida, Hongxia Liu, Shota Hori, Yuki Otsubo, Yasutake Tanaka, Masao Sato, Satoshi Ishizuka

**Affiliations:** 1 Research Faculty of Agriculture, Hokkaido University, Sapporo, 060-8589 Japan; 2 Faculty of Agriculture, Kyushu University, Fukuoka, 819-0385 Japan

**Keywords:** Sterols, Metabolomics

## Abstract

In our previous study, enterohepatic 12α-hydroxylated (12α) bile acid (BA) levels were found to be correlated with hepatic triacylglycerol concentration in rats fed high-fat (HF) diet. Since BA composition is diverse depending on animal species, we evaluated whether such a relationship is applicable in mice in response to an HF diet. C57BL/6JJmsSLC (B6) male mice were fed HF diet for 13 weeks and analyzed for triacylglycerol, cholesterol, oxysterols, and other metabolites in the liver. The BA composition was determined in the liver, small intestinal contents, portal plasma, aortic plasma, and feces. Neutral sterols were also measured in the feces. The ratio of 12α BA/non-12 BA increased in the liver, portal plasma, small intestinal contents, and feces of HF-fed B6 mice. Moreover, a positive correlation was observed between the ratio of fecal 12α BAs/non-12 BAs and hepatic triacylglycerol concentration. The concentration of 7α-hydroxycholesterol was increased in the liver of HF-fed B6 mice, whereas no increase was observed in the hepatic expression of cytochrome P450 family 7 subfamily A member 1. The present study showed that the ratio of 12α BA/non-12 BA in feces is closely associated with hepatic triacylglycerol accumulation in B6 mice fed HF diet.

## Introduction

Metabolic dysfunction-associated fatty liver disease (MAFLD) was introduced as a novel definition of hepatic disorders^1–4^. The number of patients suffering from fatty liver is increasing worldwide, and recent studies have revealed a variety of pathogeneses of fatty liver^5,6^. Nonalcoholic steatohepatitis (NASH) and hepatocellular carcinoma (HCC) have been extensively studied in the advanced phases of MAFLD pathogenesis^7,8^. It has been proposed that simple fatty liver, an initial stage of MAFLD, can be restored by improving lifestyle choices such as dietary habits and exercise^9,10^. Prevention of early phase fatty liver is expected to be a promising measure to reduce the risk of metabolic disorders such as diabetes, hepatitis, and HCC^11–13^. However, metabolic alterations that occur in the early stages have not been well studied, especially prior to the increase in triacylglycerol (TG) and cholesterol (chol) in the peripheral blood.

In our previous study^14^, a high-fat (HF) diet was found to increase the secretion of bile acids (BAs), specifically 12α-hydroxylated (12α) BAs, in the bile of rats. BA is a form of chol excretion that functions as an emulsifier for dietary lipids and contributes in their absorption^15^. The level of 12α BAs in the peripheral plasma correlates with hepatic lipid accumulation in humans^16^, and the concentration of 12α BAs in several enterohepatic tissues and fluids correlates with the hepatic TG concentration in HF-fed rats^14^. These results suggest a significant relationship between hepatic lipid accumulation and 12α BA metabolism in enterohepatic circulation.

In contrast, mice are useful for studying mechanistic aspects of metabolic disorders^17^. In particular, many genetically modified mice are available based on C57BL/6 (B6) mice using knockout or transgenic technologies. Therefore, it is necessary to clarify the relationship between BA metabolism and hepatic lipid dysregulation in mice to identify the significant events that occur in the early phase of MAFLD. In addition, some differences in BA metabolism have been observed among animal species^15^. The proportion of 12α BA in the whole BA metabolism should be considered when evaluating the association between BA metabolism and fatty liver. In this study, we investigated whether alterations in 12α BA metabolism in B6 mice fed a HF diet correlates with hepatic triacylglycerol concentration.

## Results

### Obesity induced by HF diet

In HF-fed B6 mice, there was a decrease in food intake, whereas energy intake increased (Table 1). Significant increases were observed in body weight and absolute liver weight (control: 1.2 ± 0.1 g, HF: 2.1 ± 0.1 g, *P* < 0.05, n=8) in the HF-fed B6 mice. However, the relative liver weights were comparable in both groups. Plasma ALT activity increased in HF-fed B6 mice, whereas no significant difference was observed in plasma AST activity.

**Table 1:** Food intake, weights, and plasma transaminase activities.

	C	HF
Total food intake (g)	329.5 ± 29.9	282.9 ± 12.1*
Total energy intake (kcal)	1266.0 ± 115.0	1524.9 ± 65.3*
Final body weight (g)	29.4 ± 0.8	46.5 ± 1.0*
**Organ weight (g/100 g body weight)**		
Liver	4.2 ± 0.1	4.5 ± 0.2
Epididymal adipose tissue	2.9 ± 0.2	5.3 ± 0.2*
Plasma ALT (IU/L)	3.0 ± 0.2	10.7 ± 2.4*
Plasma AST (IU/L)	65.5 ± 9.6	77.0 ± 14.8

*Significant different from the values in control (Student’s *t*-test, *P* < 0.05, n = 7–8).

### Alteration in chol metabolism

**Figure 1:**
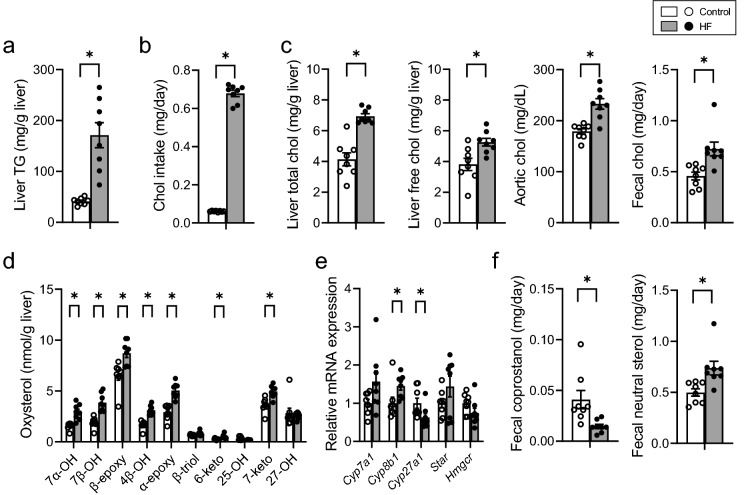
Distribution of chol-related molecules in mice fed control or HF diet. (**a**) Hepatic TG concentration. (**b**) Daily chol intake. (**c**) Chol concentration in the liver, blood, and feces. (**d**) Concentration of liver oxysterols. (**e**) mRNA expression of genes involved in chol metabolism. (**f**) Coprostanol and neutral steroid excretion per day. Open bars, n = 8 for control; filled bars, n = 8 for HF. Data presented in E was normalized to *Gapdh* mRNA expression. Values are shown as the mean ± SEM (n = 8). Asterisks indicate a significant difference compared with the control (*P* < 0.05).

A significant increase was observed in hepatic TG concentration and daily chol intake in HF-fed B6 mice (Figs. 1a,b). An increase was observed in the concentration of hepatic chol, hepatic free chol, aortic plasma chol, and fecal chol in HF-fed B6 mice (Fig. 1c). Levels of most hepatic oxysterols were found to have increased in HF-fed B6 mice (Fig. 1d). The expression of cytochrome P450 family 8 subfamily B member 1 (*Cyp8b1*), a sterol 12α-hydroxylase, was significantly increased in HF-fed B6 mice, whereas the expression of cytochrome P450 family 27 subfamily A member 1 (*Cyp27a1*), responsible for non-12 BA synthesis, was significantly decreased in HF-fed B6 mice. No change was observed in the expression of steroidogenic acute regulatory protein (*Star*) or 3-hydroxy-3-methylglutaryl-CoA reductase (*Hmgcr*) (Fig. 1e). There was a decrease in fecal coprostanol excretion in HF-fed B6 mice compared with that in the control, however, excretion of chol was increased in the HF-fed B6 mice (Fig. 1f).

### Increase in 12α BA/non-12 BA ratio in HF-fed B6 mice

**Figure 2:**
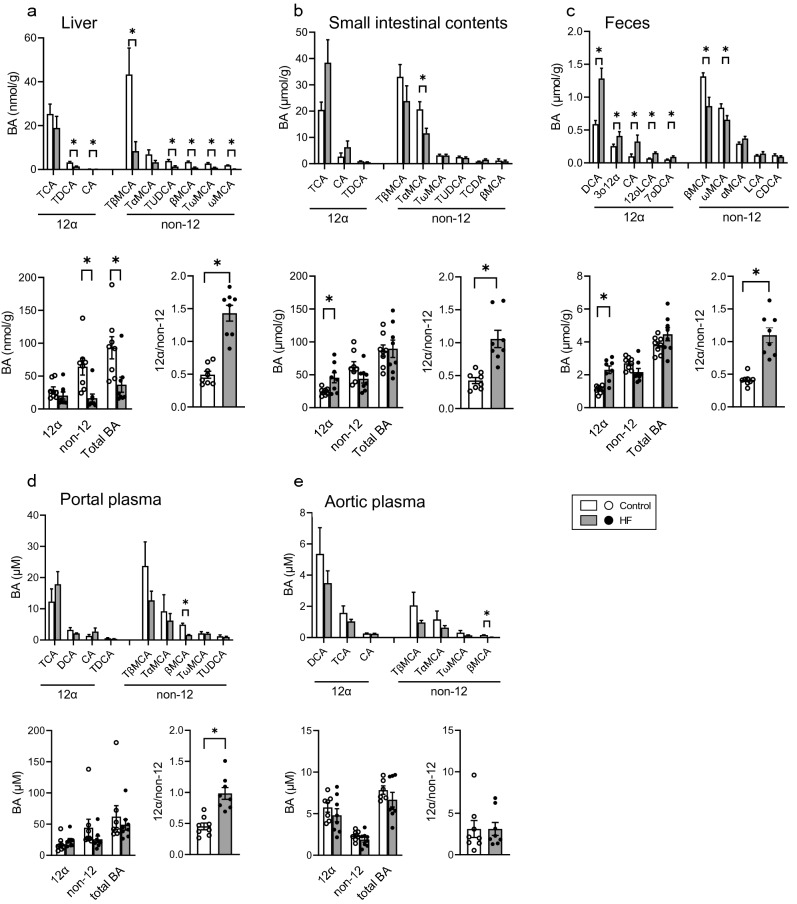
BA compositions in the liver, small intestine contents, and feces of mice fed control and HF diet. BA composition; 12α, non-12, and total BA concentrations; and the ratio of 12α BA/non-12 BA in the (**a**) liver, (**b**) small intestinal contents, (**c**) feces, (**d**) portal plasma, and (**e**) aortic plasma. Open bars represent control (n = 8) and filled bars represent HF (n = 8). Values are shown as mean ± SEM (n = 8). Asterisks indicate a significant difference compared with the control (*P* < 0.05).

As the pattern of hepatic gene expression in HF-fed B6 mice was turned to synthesize 12α BAs, we analyzed the BA composition in the liver, small intestinal contents, and feces (Fig. 2, Table S2). As shown in Fig. 2a, no difference was observed in hepatic 12α BA concentration. In contrast, a significant reduction was observed in the concentration of TβMCA, which was originally present at the highest level among the BAs in the liver. Furthermore, the concentration of non-12 BAs, including TUDCA, βMCA, TωMCA, and ωMCA, was significantly decreased in the HF-fed B6 mice (Fig. 2a). Consequently, the ratio of 12α BA/non-12 BA increased significantly, although total BA and non-12 BA concentrations in the liver were significantly decreased in HF-fed B6 mice. A similar increase in the ratio of 12α BA/non-12 BA was observed in the small intestinal contents of HF-fed B6 mice, regardless of certain minor differences, wherein TαMCA was found to have reduced in the small intestinal contents (Fig. 2b). In feces (Fig. 2c), significant increase in 12α BAs, such as DCA, 3o12α, CA, 12oLCA, and 7oDCA, was observed in the HF-fed B6 mice, whereas the concentrations of βMCA and ωMCA were significantly decreased. An increase in the ratio of 12α BA/non-12 BA was also confirmed in the feces. Such an increase in the ratio of 12α BA/non-12 BA was also observed in the portal plasma but not in the aortic plasma (Figs. 2d,e).

### Correlation between the ratio of 12α BA/non-12 BA in feces and hepatic TG concentration

**Figure 3:**
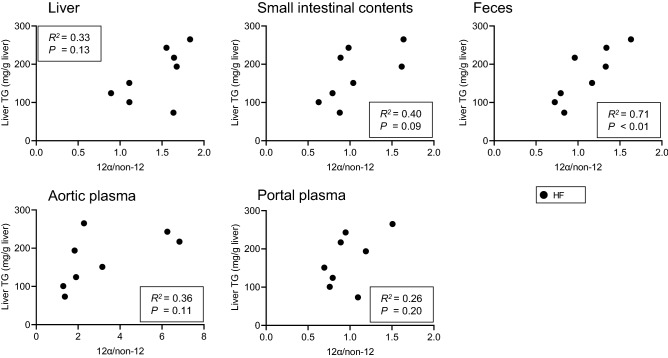
Correlation between the ratio of 12α BA/non-12 BA and hepatic TG concentration. Correlation in the liver, small intestine contents, feces, aortic plasma, and portal plasma. Filled bars represent HF (n = 8). *P*-values are shown in the inset.

The ratio of 12α BA/non-12 BA was significantly increased at several sites involved in enterohepatic circulation in HF-fed B6 mice (Fig. 2). We analyzed the correlation between the 12α BA/non-12 BA ratio and hepatic TG accumulation at the sites and organs where BA composition was measured (Fig. 3). As expected, we found a positive correlation between hepatic TG accumulation and the ratio of 12α BA/non-12 BA in the feces. In contrast, the ratio of 12α BA/non-12 BA in the liver, small intestinal contents, portal plasma and aortic plasma did not correlate with hepatic TG accumulation. We also analyzed correlation between the BA ratio and hepatic Chol (Fig. S1). There were negative correlations between total Chol and the ratio of 12α BA/non-12 BA in small intestinal contents and feces.

### Metabolome in the liver of HF-fed B6 mice

**Figure 4:**
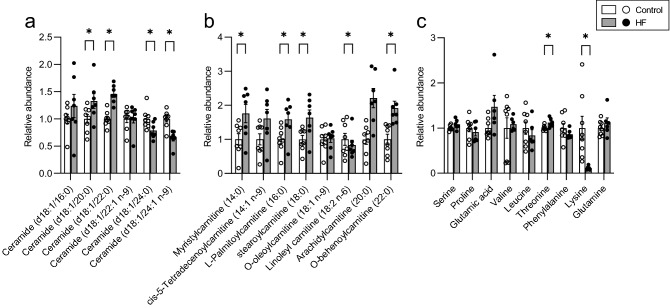
Composition of various metabolites in the liver of the mice fed control and HF diet. Relative abundances of ceramides (**a**), acylcarnitine (**b**), and amino acids (**c**). Open bars represent control (n = 8) and filled bars represent HF (n = 8). Values are shown as mean ± SEM (n = 8). Asterisks indicate a significant difference compared with the control (*P* < 0.05).

The amount of long-chain ceramides decreased, and the amount of d18:1/20:0 ceramide and d18:1/22:0 (n-9) ceramide decreased in the HF-fed B6 mice (Fig. 4a). The amount of acylcarnitine was found to have increased in many molecular species (Fig. 4b). The level of most of the amino acids remained unchanged; however, a slight increase was observed for threonine, and a significant decrease was detected for lysine (Fig. 4c). Changes were also observed in the type and concentration of fatty acids components of diacylglycerol (DG), phosphatidyl choline (PC), triacylglycerol (TG), and phosphatidyl ethanolamine (PE) (Fig. S2).

### Difference in feed efficiency between mice and rats

Feed efficiency was evaluated using data from the present mouse study and our previous rat-based analysis^14^. Feed efficiency was significantly higher in rats than that in mice, regardless of the diet composition (Fig. S3).

## Discussion

There is an enormous difference in the BA composition between tissues and feces. For example, we observed a higher fecal concentration of 12α BAs than that of non-12 BAs in humans^18^, although non-12 BAs are abundant in the peripheral plasma^19^. Likewise, the concentration of 12α BAs was high in the tissues and fluid of enterohepatic circulation in rats^20^, whereas non-12 BAs were abundant in the feces of B6 mice (Fig. 2). In a previous study, we found a positive correlation between hepatic lipid accumulation and concentration of 12α BAs in the portal plasma and feces of HF-fed rats^14^. Such a relationship between 12α BAs and hepatic lipid accumulation has been implied in some studies in mice. *Cyp8b1*-deficient mice with extremely low levels of 12α BAs showed decreased hepatic TG in mice fed a Western diet, and hepatic TG was restored when TCA was added to the diet^21^. Another study demonstrated that *Cyp8b1* deficiency ameliorates hepatic steatosis in mice fed a high-chol diet^22^. Based on these studies, we evaluated the significance of 12α BA metabolism in hepatic lipid accumulation and observed a positive association between the ratio of fecal 12α BA/non-12 BA and hepatic TG concentration in HF-fed B6 mice, suggesting that the fecal 12α BA/non-12 BA ratio is a biomarker for liver lipid accumulation in mice as well.

Such clear association with hepatic TG concentration was not observed in the liver, small intestinal contents, aortic plasma, and portal plasma (Fig. 3). A difference between feces and other sites was sampling conditions. We collected feces for 24 h in feces, but the others were collected at the end of the experiment. In other words, fecal BAs were cumulative value and the BAs in other sites were a cross-sectional value. A cumulative value may reflect the metabolic situation rather than a cross-sectional one^23^. There was no change in the concentration of 12α BA in the liver (Figs. 1e, 2a) regardless of the increase in hepatic *Cyp8b1* expression. On the other hand, 12α BA concentrations were increased in small intestinal contents and feces (Figs. 2b,c). Additionally, a negative correlation was observed between total chol and the ratio of 12α BA/non-12 BA in small intestine and feces (Fig. S1). Those observations suggests that 12α BAs synthesized in the liver were immediately secreted into the intestinal tract and excreted in the feces.

Chol contains rigid steroid backbone, which is not degraded in the body, indicating no energy can be extracted. Given that chol biosynthesis consumes certain energy and energy restriction reduces hepatic chol^24^, its synthesis can be enhanced under sufficient energy intake. However, once chol is in a sufficient amount in the body, it is converted to BAs to be discarded in water phase. 12α BAs may be one of the forms in steroid excretion. Also, such sufficient energy conditions may be involved in hepatic lipid accumulation^14^. Those point of view suggest a possibility of the correlation between hepatic TG accumulation and fecal 12α BA concentration. Such relationship may be applicable to humans. Actually, fatty liver index correlates the ratio of 12α BA/non-12 BA in aortic plasma in human^16^. There may be a more clearly correlation between hepatic TG and fecal 12α BA concentration.

Under ad libitum feeding conditions, hepatic free chol in male C57BL/6NCr mice fed a high-fat diet accounts for about 70% of total chol, while the ratio is almost reversed in females with esterified chol dominating^25^. On the other hand, it has been reported that the concentration of hepatic free chol in C57BL/6 male mice drops to about 11% of total chol when they are fed a high-fat diet and fasted overnight before dissection^26^. Thus, the ratio of free chol to esterified chol in mouse liver varies markedly depending on feeding conditions and sex. Careful attention is necessary when comparing these parameters across studies.

Compared to our rat study regarding HF diet^14^, we found some differences in the chol metabolism between mice and rats in response to the HF diet (Figs. 1, 2). In the excessive chol condition with HF diet, there is an enhancement in the conversion of chol to 12α BAs and a reduction in hepatic *Hmgcr* expression in rats^14^. On the other hand, fecal excretion of chol was enhanced in B6 mice fed the HF diet in the present study, although the concentrations of several oxysterols were increased in the liver of HF-fed mice (Figs. 1c–f) and the values were almost comparable with those in rats fed the HF diet^14^. These results suggest that mice might maintain the chol balance by excreting chol rather than by enhancing BA biosynthesis under this condition. A possible explanation for such a difference between mice and rats may stem from energy expenditure in the body. Feed efficiency was significantly lower in mice than that in rats (Fig. S3), which suggests enhanced energy expenditure in mice to maintain body temperature than in rats because of the relatively large surface area compared to the body mass in mice. In this context, if enormous amounts of carbohydrates are used to maintain the body temperature, a certain amount of chol remains in the liver. Notably, a large amount of NADPH is necessary in the liver to convert chol to BAs, regardless of the high level of energy requirement in mice^27^.

Carbohydrates rather than fatty acids may be preferentially used for energy expenditure because the oxygen requirement of carbohydrate catabolism is lower than that of fatty acid catabolism^28,29^. In this condition, the liver may discard chol in the feces in mice. Regardless of the differences in chol metabolism between mice and rats, a positive correlation was observed between the fecal 12α BA/non-12 BA ratio and hepatic TG concentration in mice. These observations suggest the significance of the fecal 12α BA/non-12 BA ratio in hepatic lipid accumulation. There was an increase in the levels of many types of acylcarnitine in HF-fed B6 mice (Fig. 4b). Lysine was specifically decreased in the liver of HF-fed B6 mice (Fig. 4c) and is a substrate for carnitine biosynthesis^30–32^. The decrease in lysine could be due to accelerated carnitine biosynthesis in the liver. Carnitine promotes β-oxidation of fatty acids via the transport of long-chain fatty acids into in mitochondria^33^. An increase in carnitine levels enables hepatocytes to catabolize fatty acids in response to excessive fat. However, there was no difference in the expression of gamma-butyrobetaine hydroxylase 1 (*Bbox1*) (data not shown), the rate-limiting enzyme for carnitine biosynthesis, despite an increase observed in the corresponding protein level in HF-fed rats^34,35^. The amount of BBOX1 increases in HF-fed rats through increased polyadenylation efficiency, mRNA stabilization, and translation efficiency^35^. These observations suggest an increase in the amount of BBOX1 protein in the present study.

Certain alterations were observed in the fatty acid composition of TG, DG, PC, PE, and PS in the metabolomic analysis; however, no significant pathway was identified during the pathway analysis. This may be due to the broad spectrum of lipid characteristics, particularly hydrophilicity. The extraction solvent is diverse depending on the lipid of interest, which suggests a limitation in the comprehensive analysis of lipids in terms of extraction efficiency compared with hydrophilic molecules. In this study, the free chol concentration was also analyzed in the lipid samples extracted for lipidomics, and no significant difference was observed between the groups (data not shown). In contrast, there was a significant increase in free chol in the liver of the mice fed the HF diet, when extracted using Folch’s method^36^ (Fig. 1c). In general, lipidomic analysis is undoubtedly useful for estimating alterations in lipid composition, but it is to be noted that there is no gold standard for the extraction of every molecular species in lipids. The appropriate selection of organic solvents in combination with careful evaluation is necessary, especially in the application of lipidomic analysis.

In conclusion, a positive correlation was observed between the fecal 12α BA/non-12 BA ratio and hepatic lipid accumulation in B6 mice fed an HF diet. Regardless of difference in chol metabolism between mice and rats, fecal BAs can be used as a marker for hepatic TG concentration in mice.

## Methods

### Animals and diets

Animal experiments were approved by the Institutional Animal Care and Use Committee of the National Corporation Hokkaido University (approval numbers: 17-0119 and 19-0161), and all animals were maintained in accordance with the Hokkaido University Manual for Implementing Animal Experimentation. The study was carried out in compliance with the ARRIVE guidelines. C57BL/6JJmsSlc mice (4 weeks old; male) were purchased from Japan SLC, Inc. (Hamamatsu, Japan). The mice were kept in separate cages with a 12-h light/dark cycle (light period: 8:00–20:00) at 22 ± 2 °C and 55 ± 5% humidity. The mice had access to food and water ad libitum. After 1 week of acclimation to the control diet, the mice were divided into two dietary groups fed either a control or HF diet (n = 8 each) and maintained for 13 weeks. The dietary compositions are shown in Table S1. At week 13, mice were intraperitoneally injected with sodium pentobarbital (50 mg/kg body weight; Kyoritsu Seiyaku Corporation, Tokyo, Japan) and anesthetized. Heparin sodium (Yoshindo Inc., Toyama, Japan) was added to the blood withdrawn from the portal vein and heart. Plasma was collected and stored at −80 °C for lipid, transaminase, and BAs analyses. The liver and epididymal adipose tissue were weighed and the liver was stored at −80 °C for lipid and gene expression analyses. The small intestinal contents were squeezed out and collected. The liver, small intestinal contents, blood plasma, and feces were stored at −30 °C for BA analysis.

### Measurement of the biochemical parameters

Lipids were extracted from the liver and feces according to the method described by Folch^36^ and re-dissolved in isopropanol for measurement after evaporation. TG, chol, and free chol levels were determined in the liver using Triglyceride E-test, Cholesterol E-test, and Free cholesterol test, (FUJIFILM Wako Pure Chemical Corporation, Osaka, Japan), respectively. Chol was also measured in the blood plasma and feces. Fecal neutral sterols were derivatized to trimethylsilyl ethers and measured using gas–liquid chromatography^34^. Plasma alanine aminotransferase (ALT) and aspartate aminotransferase (AST) activities were measured using the transaminase C-II test Wako kit (FUJIFILM Wako Pure Chemical).

### BA analysis

BAs were measured according to the method described by Hori et al^14^. Each tissue extract was purified by solid-phase extraction using HLB 1 cc (10 mg) extraction cartridges and separated with an ultra-high-performance liquid chromatograph Ultimate 3000 (Thermo Fisher Scientific K.K., Tokyo, Japan) equipped with an ACQUITY UPLC BEH C18 1.7 μm column (Nihon Waters K. K., Tokyo, Japan). BAs were detected using Q Exactive Plus Hybrid Quadrupole-Orbitrap Mass Spectrometer (Thermo Fisher Scientific). 23-Nor-5β-cholanic acid was used as the internal standard.

### Gene expression analysis

Total RNA was isolated from the liver using RNeasy Mini Kit (Qiagen, Hilden, Germany) according to the manufacturer’s instructions. Complementary DNA was synthesized using ReverTra Ace RT master mix with gDNA remover (TOYOBO Co., Osaka, Japan). Quantitative RT-PCR was carried out using the SYBER Green method using SYBER Premix Ex Taq II Green (Takara Bio Inc., Kusatsu, Japan) and Mx3000P real-time PCR system (Agilent Technologies Japan, Ltd. Tokyo, Japan). The primers used for qRT-PCR are listed in Table S3. mRNA expression data were normalized using glyceraldehyde-3-phosphate dehydrogenase (*Gapdh*) expression.

### Oxysterol analysis

The oxysterol concentration was measured according to previous reports^14,37^. Briefly, lipids were extracted using chloroform/methanol (2:1, v/v) containing butylated hydroxytoluene. After overnight saponification, unsaponified lipids were extracted with hexane. The extracted lipids were applied to a Sep-Pak Silica Vac cartridge (Nihon Waters) to separate oxysterols from chol. After evaporating the oxysterol-containing solvent fraction under N_2_, dried residues were converted into trimethylsilyl ethers. Oxysterol was quantified by gas chromatography–mass spectrometry using a Shimadzu GC-2010 Plus instrument (Shimadzu Corporation, Kyoto, Japan) coupled with an Inert Cap 5MS/NP capillary column (30 m × 0.25 mm i.d., 0.25 μm thick, GL Sciences Inc, Tokyo, Japan.) connected to a QP2020 series mass-selective detector (Shimadzu). The concentrations of individual oxysterols were measured using 19-hydroxycholesterol (Steraloids, Inc. Newport, RI, USA) as the internal standard. The oxysterols analyzed in this study are listed in Table S4.

### Metabolomic analysis

Extraction for metabolomics was based on the method reported by Wu et al^38^. with some modifications. Approximately 50 mg of the liver was weighed at the time of dissection, frozen in liquid nitrogen, and stored at −80 °C. An extraction solution (4 mL/g sample of cold methanol and 0.85 mL/g sample of cold ultrapure water) was added to the sample. The mixture was homogenized on ice and sonicated. Chloroform (4 mL/g sample) and ultrapure water (4.4 mL/g sample) were added to the sonicated samples and mixed using a vortex for 60 s. The homogenates were then left on ice for 10 min and centrifuged at 2000 × g for 5 min to produce a biphasic mixture. Aqueous (the upper layer) and organic (the lower layer) extracts were collected separately. Each extract was filtered through a Millex 0.2 μm filter (Merck, Darmstadt, Germany). The aqueous extracts were dried using a centrifugal concentrator VC-96 N with a freeze trap VA-250F (Taitec Co., Saitama, Japan), and the organic extracts were dried using nitrogen gas. Both extracts were stored at −80 °C until analysis. The aqueous extracts for amino acid analysis were resuspended in 1 mL of methanol/water (50:50, v/v) and the organic extracts for lipid analysis were resuspended in 1 mL of acetonitrile/isopropanol/water (60:30:5, v/v/v). The aqueous and organic extracts were analyzed separately using an Ultimate 3000 and Q Exactive Plus Hybrid Quadrupole-Orbitrap Mass Spectrometer (Thermo Fisher Scientific). For amino acid analysis, chromatography was carried out at 50 °C on a SeQuant ZIC-pHILIC Column (5 µm, 2.1 × 150 mm, Merck) with the following mobile phase: *A* = 10 mM ammonium acetate in 5% acetonitrile, *B* = 10 mM ammonium acetate in 95% acetonitrile. A gradient was used at a flow rate of 0.2 mL/min: 99−50% of B from 0 to 10 min, 50% of B from 10 to 14.5 min, 50−99% of B from 14.5 to 15 min, and 99% of B from 15 to 20 min. The injection volume was 3 µL. For lipid analysis, chromatography was carried out at 60 °C on a Thermo Hypersil GOLD Column (1.9 µm, 2.1 × 150 mm, Thermo Fisher) with the following mobile phase: *A* = 10 mM ammonium formate in 60% acetonitrile, *B* = 10 mM ammonium formate in acetonitrile/isopropanol (10:90, v/v). A gradient was used at 0.3 mL/min: 30% of B from 0 to 2 min, 30−60% of B from 2 to 6 min, 60−100% of B from 6 to 11 min, 100% of B from 11 to 13 min, 100−30% of B from 13 to 13.5 min, and 30% of B from 13.5 to 15 min. The injection volume was 2 µL. The MS settings were as follows: mass range was set from 67 to 1000 m/z for amino acid analysis and 200 to 1450 m/z for lipid analysis; spray voltage, 2.5 kV; capillary temp, 250 °C; sheath gas flow rate, 45 (arbitrary units); aux gas flow rate, 10 (arbitrary units); aux gas heat temp, 400 °C; resolution 1.4e5; AGC target 1e6. ESI was operated in a positive/negative dual polarity mode for sample analysis. The data obtained were evaluated using Compound Discoverer 3.0 software (Thermo Fisher Scientific).

### Statistics

All data are depicted as means ± SEM. A significant difference test was performed using JMP Pro15 (SAS Institute Inc., Cary, NC, USA). Two group comparison was determined by Student’s *t*-test and differences were considered significant when the *P*-value was less than 0.05. Pearson’s method was used to evaluate the correlations. Smirnov-Grubbs’ test was used to determine the outliers.

## Supplementary Information

Supplementary Information.

## Data Availability

The datasets generated and analyzed during the current study are available from the corresponding author on reasonable request.

## Abbreviations

12α: 12α-Hydroxylated
7αOH: 7α Hydroxycholesterol
ALT: Alanine aminotransferase
AST: Asparate aminotransferase
B6: C57BL/6 J
BA: Bile acid
BBOX1: Gamma-butyrobetaine hydroxylase 1
CA: Cholic acid
Chol: Cholesterol
CYP27A1: Cytochrome P450 family 27 subfamily A member 1
CYP7A1: Cytochrome P450 family 7 subfamily A member 1
CYP8B1: Cytochrome P450 family 8 subfamily B member 1
DG: Diacylglycerol
GAPDH: Glyceraldehyde-3-phosphate dehydrogenase
HCC: Hepatocellular carcinoma
HF: High-fat
HMGCR: 3-Hydroxy-3-methylglutaryl-CoA reductase
MAFLD: Metabolic dysfunction-associated fatty liver disease
NASH: Nonalcoholic steatohepatitis
PC: Phosphatidyl choline
PE: Phosphatidyl ethanolamine
PS: Phosphatidyl serine
qRT-PCR: Quantitative reverse transcription polymerase chain reaction
STAR: Steroidogenic acute regulatory protein
TG: Triacylglycerol

## References

[1] Eslam M (2020). A new definition for metabolic dysfunction-associated fatty liver disease: An international expert consensus statement. J. Hepatol..

[2] Eslam M, Sanyal AJ, George J (2020). MAFLD: A consensus-driven proposed nomenclature for metabolic associated fatty liver disease. Gastroenterology.

[3] Lee H, Lee YH, Kim SU, Kim HC (2021). Metabolic dysfunction-associated fatty liver disease and incident cardiovascular disease risk: A nationwide cohort study. Clin. Gastroenterol Hepatol..

[4] Nguyen VH, Le MH, Cheung RC, Nguyen MH (2021). Differential clinical characteristics and mortality outcomes in persons with NAFLD and/or MAFLD. Clin. Gastroenterol Hepatol..

[5] Parthasarathy G, Revelo X, Malhi H (2020). Pathogenesis of nonalcoholic steatohepatitis: An overview. Hepatol. Commun..

[6] Younossi ZM (2019). Non-alcoholic fatty liver disease–a global public health perspective. J. Hepatol..

[7] Kanwal F (2018). Risk of hepatocellular cancer in patients with non-alcoholic fatty liver disease. Gastroenterology.

[8] Dongiovanni P, Meroni M, Longo M, Fargion S, Fracanzani AL (2021). Genetics, immunity and nutrition boost the switching from NASH to HCC. Biomedicines..

[9] Glass O (2020). Standardisation of diet and exercise in clinical trials of NAFLD-NASH: Recommendations from the liver forum. J. Hepatol..

[10] Younossi ZM, Corey KE, Lim JK (2021). AGA clinical practice update on lifestyle modification using diet and exercise to achieve weight loss in the management of nonalcoholic fatty liver disease: Expert review. Gastroenterology.

[11] Chrysavgis L, Giannakodimos I, Diamantopoulou P, Cholongitas E (2022). Non-alcoholic fatty liver disease and hepatocellular carcinoma: Clinical challenges of an intriguing link. World J. Gastroenterol..

[12] Perry RJ, Samuel VT, Petersen KF, Shulman GI (2014). The role of hepatic lipids in hepatic insulin resistance and type 2 diabetes. Nature.

[13] Geisler CE, Renquist BJ (2017). Hepatic lipid accumulation: cause and consequence of dysregulated glucoregulatory hormones. J. Endocrinol..

[14] Hori S (2020). Association between 12α-hydroxylated bile acids and hepatic steatosis in rats fed a high-fat diet. J. Nutr. Biochem..

[15] de Aguiar Vallim TQ, Tarling EJ, Edwards PA (2013). Pleiotropic roles of bile acids in metabolism. Cell Metab..

[16] Haeusler RA, Astiarraga B, Camastra S, Accili D, Ferrannini E (2013). Human insulin resistance is associated with increased plasma levels of 12α-hydroxylated bile acids. Diabetes.

[17] Zhong F, Zhou X, Xu J, Gao L (2020). Rodent models of nonalcoholic fatty liver disease. Digestion.

[18] Hashimoto N (2020). Lithocholic acid increases intestinal phosphate and calcium absorption in a vitamin D receptor dependent but transcellular pathway independent manner. Kidney Int..

[19] Jiao N (2018). Suppressed hepatic bile acid signaling despite elevated production of primary and secondary bile acids in NAFLD. Gut.

[20] Lee J-Y (2020). 12α-Hydroxylated bile acid induces hepatic steatosis with dysbiosis in rats. Biochim. Biophys. Acta. Mol. Cell. Biol. Lipids..

[21] Bertaggia E (2017). *Cyp8b1* ablation prevents Western diet-induced weight gain and hepatic steatosis because of impaired fat absorption. Am. J. Physiol. Endocrinol Metab..

[22] Patankar JV (2018). Genetic ablation of *Cyp8b1* preserves host metabolic function by repressing steatohepatitis and altering gut microbiota composition. Am. J. Physiol. Endocrinol Metab..

[23] Cerqueira NM (2016). Cholesterol biosynthesis: A mechanistic overview. Biochemistry.

[24] Rocha-Gomes A (2021). Caloric restriction or cafeteria diet from birth to adulthood increases the sensitivity to ephedrine in anxiety and locomotion in Wistar rats. Physiol. Behav..

[25] Milligan S (2018). Ablating both *Fabp1* and *Scp2/Scpx* (TKO) induces hepatic phospholipid and cholesterol accumulation in high fat-fed mice. Biochim. Biophys. Acta Mol. Cell. Biol. Lipids.

[26] Hoekstra M (2021). SR-BI deficiency disassociates obesity from hepatic steatosis and glucose intolerance development in high fat diet-fed mice. J. Nutr. Biochem..

[27] Dashty M (2013). A quick look at biochemistry: Carbohydrate metabolism. Clin. Biochem..

[28] Houten SM (2016). The biochemistry and physiology of mitochondrial fatty acid β-oxidation and its genetic disorders. Annu. Rev. Physiol..

[29] Horne DW, Broquist HP (1973). Role of lysine and ε-N-trimethyllysine in carnitine biosynthesis. I. Studies in neurospora crassa. J. Biol. Chem..

[30] Tanphaichitr V, Broquist HP (1988). Role of lysine and ε-N-trimethyllysine in carnitine biosynthesis. II. Studies in the rat. Nutr. Rev..

[31] Vaz FM, Wanders RJ (2002). Carnitine biosynthesis in mammals. Biochem. J..

[32] Kunau WH, Dommes V, Schulz H (1995). beta-oxidation of fatty acids in mitochondria, peroxisomes, and bacteria: A century of continued progress. Prog. Lipid Res..

[33] Ling B, Aziz C, Alcorn J (2012). Systematic evaluation of key L-carnitine homeostasis mechanisms during postnatal development in rat. Nutr. Metab. (Lond).

[34] Rigault C, Le Borgne F, Tazir B, Benani A, Demarquoy J (2013). A high-fat diet increases L-carnitine synthesis through a differential maturation of the Bbox1 mRNAs. Biochim. Biophys. Acta..

[35] Folch J, Lees M, Sloane Stanley GH (1957). A simple method for the isolation and purification of total lipides from animal tissues. J. Biol. Chem..

[36] Grundy SM, Ahrens EH, Miettinen TA (1965). Quantitative isolation and gas-liquid chromatographic analysis of total fecal bile acids. J. Lipid Res..

[37] Shirouchi B (2017). 27-Hydroxycholesterol suppresses lipid accumulation by down-regulating lipogenic and adipogenic gene expression in 3T3-L1 cells. Cytotechnology.

[38] Wu H, Southam AD, Hines A, Viant MR (2008). High-throughput tissue extraction protocol for NMR- and MS-based metabolomics. Anal. Biochem..

