# Author Correction: The ratio of 12α to non-12-hydroxylated bile acids reflects hepatic triacylglycerol accumulation in high-fat diet-fed C57BL/6J mice

**DOI:** 10.1038/s41598-022-26541-z

**Published:** 2022-12-20

**Authors:** Wakana Iwasaki, Ryo Yoshida, Hongxia Liu, Shota Hori, Yuki Otsubo, Yasutake Tanaka, Masao Sato, Satoshi Ishizuka

**Affiliations:** 1 Research Faculty of Agriculture, Hokkaido University, Sapporo, 060-8589 Japan; 2 Faculty of Agriculture, Kyushu University, Fukuoka, 819-0385 Japan

Correction to: *Scientific Reports* 10.1038/s41598-022-20838-9, published online 06 October 2022

The original version of this Article contained an error in Figure 1e, where the X-axis label *Cyp27a1* was incorrectly stated as *Cyp27b1*.

The original Figure 1 and accompanying legend appear below.

**Figure 1:**
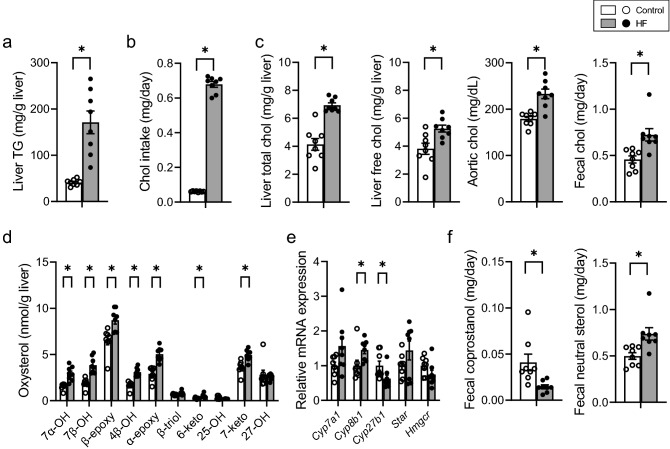
Distribution of chol-related molecules in mice fed control or HF diet. (**a**) Hepatic TG concentration. (**b**) Daily chol intake. (**c**) Chol concentration in the liver, blood, and feces. (**d**) Concentration of liver oxysterols. (**e**) mRNA expression of genes involved in chol metabolism. (**f**) Coprostanol and neutral steroid excretion per day. Open bars, n = 8 for control; filled bars, n = 8 for HF. Data presented in E was normalized to *Gapdh* mRNA expression. Values are shown as the mean ± SEM (n = 8). Asterisks indicate a significant difference compared with the control (*P* < 0.05).

Additionally, the Supplementary Information Table S2 published with this Article contained an error. Under the subheading

‘12α-hydroxylated BAs’, ‘Secondary BAs’,

“5β-cholanic acid-3α,12α-diol-7-one (7-uxo-deoxycholic acid, 7oDCA)”.

now reads:

“5β-cholanic acid-3α,12α-diol-7-one (7-oxo-deoxycholic acid, 7oDCA)”.

The original Article and accompanying Supplementary Information file have been corrected.

