# Supplementary material for: The ratio of 12α to non-12-hydroxylated bile acids reflects hepatic triacylglycerol accumulation in high-fat diet-fed C57BL/6J mice
Source: Sci Rep. 2022 Oct 6;12:16707. doi: 10.1038/s41598-022-20838-9 (PMC9537321; doi:10.1038/s41598-022-20838-9)
Supplement: Supplementary file 1 — Supplementary Information. [file 41598_2022_20838_MOESM1_ESM.pdf]

Table S1. Diet compositions

	C	HF
	g/kg diet	
Corn starch [1]	397.5	167.5
Casein [2]	200.0	200.0
Dextrin [3]	132.0	132.0
Sucrose [4]	100.0	100.0
Soybean oil [5]	70.0	70.0
Cellulose [6]	50.0	50.0
Mineral mixture [7]	35.0	35.0
Vitamin mixture [8]	10.0	10.0
L-Cystine [9]	3.0	3.0
Choline hydrogen tartrate [10]	2.5	2.5
Lard [11]	-	230.0

[1] Amylalpha (Chuo Shokuryo Co., Ltd.)
[2] NZMP Acid Casein (Fonterra Co-Operative Group Limited.)
[3] TK-16 (Matsutani Chemical Industry Co., Ltd.)
[4] Nippon Beet Sugar Manufacturing Co., Ltd.
[5] J-Oil Mills, Inc.
[6] Microcrystalline cellulose (Ceolus PH-102, Asahi Kasei Corporation)
[7] AIN-93G mineral mixture (MP Biomedicals)
[8] AIN-93 vitamin mixture (CLEA Japan, Inc.)
[9] L-Cystine (Fujifilm Wako Pure Chemical Corporation)
[10] Choline Hydrogen Tartrate (Fujifilm Wako Pure Chemical Corporation)
[11] Bell Shokuhin Co., Ltd.

Table S2. BAs analyzed in this study

12α-hydroxylated BAs
Primary BAs
5β-cholanic acid-3α,7α,12α-triol (cholic acid, CA)
5β-cholanic acid-3α,7α,12α-triol-*N*-(2-sulfoethyl)-amide (taurocholic acid, TCA)
5β-cholanic acid-3α,7α,12α-triol-*N*-(carboxymethyl)-amide (glycocholic acid, GCA)
Secondary BAs
5β-cholanic acid-3α,12α-diol (deoxycholic acid, DCA)
5β-cholanic acid-3α,12α-diol-*N*-(2-sulfoethyl)-amide (taurodeoxycholic acid, TDCA)
5β-cholanic acid-3α,12α-diol-*N*-(carboxymethyl)-amide (glycodeoxycholic acid, GDCA)
5β-cholanic acid-3α,7β,12α-triol (ursocholic acid, UCA)
5β-cholanic acid-3α,12α-diol-7-one (7-oxo-deoxycholic acid, 7oDCA)
5β-cholanic acid-3α-ol-12-one (12-oxo-lithocholic acid, 12oLCA)
5β-cholanic acid-12α-ol-3-one (3-oxo-12α-hydroxy-5β-cholanic acid, 3o12α)
Non-12-hydroxylated BAs
Primary BAs
5β-cholanic acid-3α,7α-diol (chenodeoxycholic acid, CDCA)
5β-cholanic acid-3α,6α,7α-triol (hyocholic acid, HCA)
5β-cholanic acid-3α,6β,7α-triol (α-muricholic acid, αMCA)
5β-cholanic acid-3α,6β,7β-triol (β-muricholic acid, βMCA)
5β-cholanic acid-3α,7α-diol-*N*-(2-sulfoethyl)-amide (taurochenodeoxycholic acid, TCDCA)
5β-cholanic acid-3α,7α-diol-*N*-(carboxymethyl)-amide (glycochenodeoxycholic acid, GCDCA)
5β-cholanic acid-3α,6β,7α-triol-*N*-(2-sulfoethyl)-amide (tauro-α-muricholic acid, TαMCA)
5β-cholanic acid-3α,6β,7β-triol-*N*-(2-sulfoethyl)-amide (tauro-β-muricholic acid, TβMCA)
Secondary BAs
5β-cholanic acid-3α,6α,7β-triol (ω-muricholic acid, ωMCA)
5β-cholanic acid-3α,6α,7β-triol-*N*-(2-sulfoethyl)-amide (tauro-ω-muricholic acid, TωMCA)
5β-cholanic acid-3α,7β-diol (ursodeoxycholic acid, UDCA)
5β-cholanic acid-3α,7β-diol-*N*-(2-sulfoethyl)-amide (tauroursodeoxycholic acid, TUDCA)
5β-cholanic acid-3α,7β-diol-*N*-(carboxymethyl)-amide (glycoursodeoxycholic acid, GUDCA)
5β-cholanic acid-3α-ol (lithocholic acid, LCA)
5β-cholanic acid-3α-ol-*N*-(2-sulfoethyl)-amide (taurolithocholic acid, TLCA)
5β-cholanic acid-3α-ol-*N*-(carboxymethyl)-amide (glycolithocholic acid, GLCA)
5β-cholanic acid-3α-ol-7-one (7-oxo-lithochoic acid, 7oLCA)
5β-cholanic acid-3α,6α-diol (hyodeoxycholic acid, HDCA)
5β-cholanic acid-3α,6α-diol-*N*-(2-sulfoethyl)-amide (taurohyodeoxycholic acid, THDCA)
5β-cholanic acid-3α,6α-diol-*N*-(carboxymethyl)-amide (glycohyodeoxyxholic acid, GHDCA)

Each name was listed as systemic name with synonym and abbreviation. All BA standards analyzed in this study were obtained from Steraloids, Inc., except for UCA, which was obtained from Toronto Research Chemicals.

Table S3. Primer sequences for qRT-PCR

Gene		sequence (5'- 3')	NCBI reference sequence
Cyp7a1	Forward	CCTGCAACCTTCTGGAGCTTA	NM_007824.3
	Reverse	AGCCTCCTTGATGATGCTATCTAGT	
Cyp8b1	Forward	ATGAGCTGTTCAGGAAGTTC	NM_010012.3
	Reverse	TGTCCTGCATGGATGAAGC	
Cyp27a1	Forward	CCAATGTGGACAACCTCCT	NM_024264.5
	Reverse	CTTGTGGTCTCGGTGGTC	
Star	Forward	GGAGCTCTCTGCTTGGTTCTC	NM_011485.5
	Reverse	ACCTCCAAGCGAAACACCTT	
Gapdh	Forward	TGACCTCAACTACATGGTCTACA	NM_001289726.1
	Reverse	CTTCCCATTCTCGGCCTTG	
Bbox1	Forward	TTCTCAACAGGCCAGAGCAA	NM_130452.1
	Reverse	TTCAGAGTTGGCAGCTGGAG	

Table S4. Oxysterols analyzed in this study

5-cholesten-3β,4β-diol (4β-hydroxycholesterol, 4βOH)
5α-cholestan-3β-ol-6-one (6-ketocholestanol, 6keto)
5-cholesten-3β-ol-7-one (7-ketocholesterol, 7keto)
5-cholesten-3β,7α-diol (7α-hydroxycholesterol, 7αOH)
5-cholesten-3β,7β-diol (7β-hydroxycholesterol, 7αOH)
cholestan-5α,6α-epoxy-3β-ol (5α-epoxycholesterol, α-epoxy)
cholestan-5β,6β-epoxy-3β-ol (5β-epoxycholesterol, β-epoxy)
cholestane-3β,5α,6β-triol (β-triol)
5-cholesten-3β,25-diol (25-hydroxycholesterol, 25OH)
25*R*-cholest-5-en-3β,26-diol (27-hydroxycholesterol, 27OH)
5-cholestene-3β,22(*R*)-diol (22(*R*)-hydroxycholesterol)
5-cholesten-3β,7α,25-triol (7α, 25(*R*)-dihydroxycholesterol)
5-cholesten-3β,24(*S*)-diol (24(*S*)-hydroxycholesterol)

Each name was listed as systemic name with synonym and abbreviation if necessary.

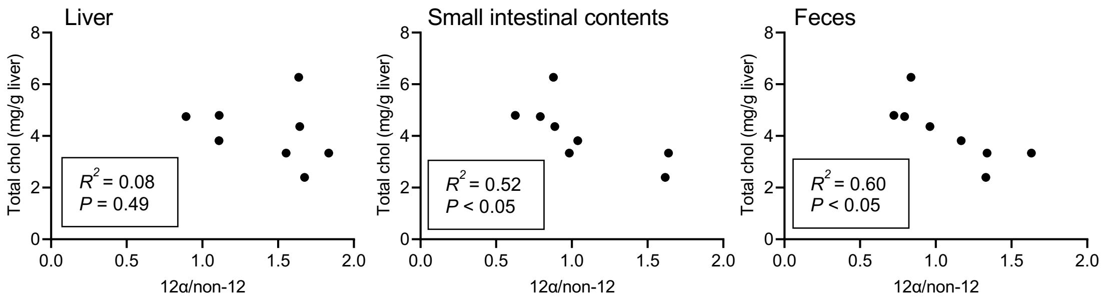

Figure S1. Correlation between the ratio of 12α BA/non-12 BA and hepatic total chol (Total chol) concentration. Correlation in the liver, small intestine contents, feces, aortic plasma, and portal plasma. Filled bars represent HF (n = 8). *P*-values are shown in the inset.

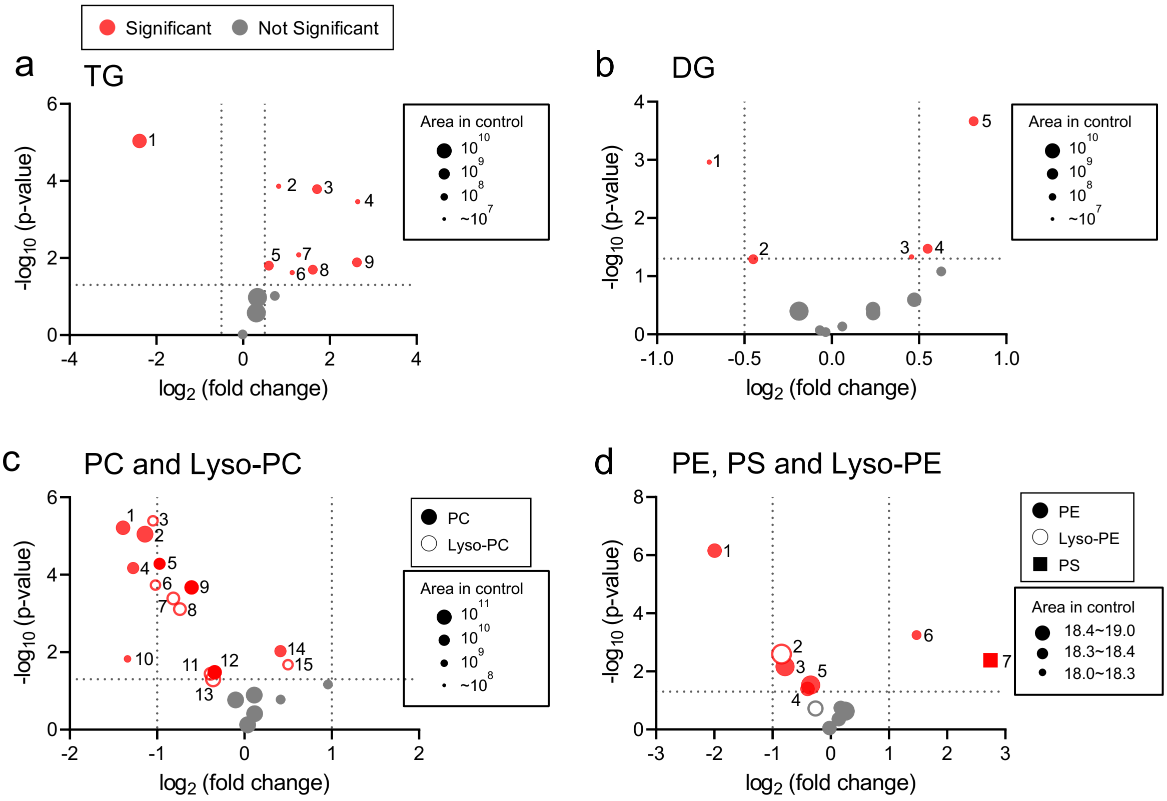

Figure S2. Composition of various metabolites in liver of the mice fed control and HF diet.

(a) Triacylglycerol (TG); (1) 20:0/20:1 (n-9)/20:4 (n-6), (2) 18:2 (n-6)/18:2 (n-6)/18:2 (n-6), (3) 16:1 (n-7)/18:1 (n-9)/18:1 (n-9), (4) 16:0/18:2 (n-6)/18:2 (n-6), (5) 17:1 (n-8)/20:2 (n-6)/20:2 (n-6), (6) 18:4 (n-3)/19:4 (n-6)/18:4 (n-3), (7) 18:3 (n-3)/14:0/18:3 (n-3), (8) 18:1 (n-9)/18:2 (n-6)/18:1 (n-9), (9) 18:2 (n-6)/18:2 (n-6)/20:2 (n-6). (b) Diacylglycerol (DG); (1) 18:2 (n-6)/18:2 (n-6), (2)18:3 (n-3)/20:3 (n-6), (3) 14:0/14:1 (n-5), (4) 15:0/16:1 (n-7), (5) 16:1 (n-7)/18:1 (n-9). (c) Phosphatidyl choline (PC) and lyso-PC; (1) 18:3 (n-3)/18:2 (n-6) PC, (2) 16:0/18:3 (n-3) PC, (3) 16:1 (n-7) lyso-PC, (4) 18:3 (n-6)/p-18:1 (n-7) PC, (5) p-16:0/16:0 PC, (6) 22:5 (n-3) lyso-PC, (7) 20:4 (n-6) lyso-PC, (8) 18:2 (n-6) lyso-PC, (9) 14:0/18:1 (n-9) PC, (10) 20:5 (n-3)/24:1 (n-9) PC, (11) 18:1 (n-9) lyso-PC, (12) 16:0/18:0 PC, (13) 16:0 lyso-PC, (14) 18:3 (n-3)/18:3 (n-3) PC, (15) 17:0 lyso-PC. (d) Phosphatidyl ethanolamine (PE), phosphatidyl serine (PS), and lyso-PE (1) 20:4 (n-6)/22:6 (n-3) PE, (2) 22:6 (n-3) lyso-PE, (3) 18:1 (n-7)/20:4 (n-6) PE, (4) 18:1 (n-9)/18:2 (n-6) PE, (5) 18:4 (n-3)/24:1 (n-9) PE, (6) 18:2 (n-6)/24:0 PE, (7) 24:0/24:0 PS. Each marker size indicates range of area values shown in inset. Markers in red indicate a significant difference between the dietary groups (n = 8, $P < 0.05$).

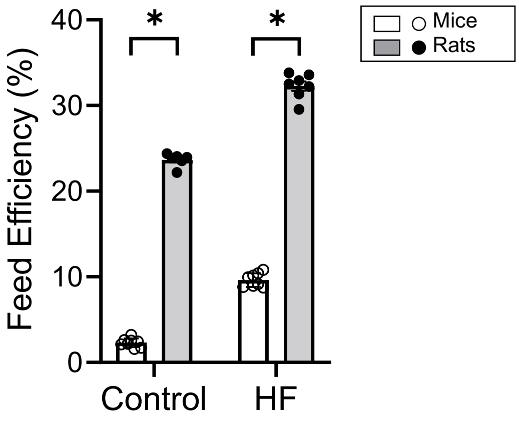

Figure S3. Difference in feed efficiency between mice and rats
The ratio of weight gain to food intake over 8 weeks was calculated. The values in rats were calculated with the data in our previous study[14].

$$\text{Feed Efficiency (\%)} = \frac{\text{Weight gain (g)}}{\text{Total food intake (g)}} \times 100$$

Open bars for mice (n = 8) and filled bars for rats (n = 6-7). Values were shown as the mean with the SEM. Asterisks indicate a significant difference compared to control ($P < 0.05$).
